# Comparative Effectiveness of Lobectomy, Segmentectomy, and Wedge Resection for Pathological Stage I Non-small Cell Lung Cancer in Elderly Patients: A Population-Based Study

**DOI:** 10.3389/fsurg.2021.652770

**Published:** 2021-04-15

**Authors:** Xining Zhang, Gang Lin, Jian Li

**Affiliations:** Department of Thoracic Surgery, Peking University First Hospital, Beijing, China

**Keywords:** elderly, limited resection, lobectomy, non-small cell lung cancer, surgery

## Abstract

**Introduction:** This study was designed to assess the long-term survival of lobectomy, segmentectomy, and wedge resection for pathological stage I non-small cell lung cancer (NSCLC) in patients over 75 years of age.

**Patients and methods:** Pathological stage I NSCLC patients aged ≥75 years who underwent lobectomy, segmentectomy, or wedge resection were identified from the Surveillance, Epidemiology, and End Results database. Propensity score–matched and competing risks analyses were conducted. The overall survival (OS) rate and lung cancer–specific survival (LCSS) rate were compared among the three groups based on the pathological stage.

**Results:** A total of 3,345 patients were included. In the full cohort, the OS rate and LCSS rate of lobectomy were superior to wedge resection, but not to segmentectomy, the OS advantage diminished when patients were over 85 years old or when at least one lymph node was examined during the procedure. Stratified analyses showed that there was no significant difference in OS and LCSS rates among the three surgical procedures for patients with tumors smaller than 1.0 cm. The OS and LCSS of wedge resection, not segmentectomy, were inferior to lobectomy in stage IA2–IB tumors.

**Conclusion:** Lobectomy should be recognized as the “gold standard” procedure for pathological stage I NSCLC in patients over 75 years of age, and segmentectomy could be considered as an effective alternative. Wedge resection could be considered for patients with compromised cardiopulmonary function or tumors smaller than 1.0 cm, and intraoperative lymph node examination should be conducted.

## Introduction

Although the lung cancer mortality rate has been declining since its peak of 215.1 deaths (per 100,000 population) in 1991, it is estimated that lung cancer will still be responsible for almost one-quarter of all cancer deaths by year 2020 (1). Meanwhile, the number of adults aged ≥65 years is expected to increase to 72 million by 2030 in the USA, and a 67% increase in incidence was projected for lung cancer (2). Furthermore, due to underrepresentation in cancer clinical trials, the elderly represent an important population that may be particularly vulnerable to suboptimal cancer care (3, 4). In addition, with the application of low-dose CT (LDCT)–based lung cancer screening programs worldwide (5–7), more early-stage lung cancer will be discovered in the future. Therefore, it is urgent to seek appropriate treatment strategies for early-stage NSCLC in the elderly patients.

The current National Comprehensive Cancer Network (NCCN) guideline for NSCLC recommended surgical resection over other local treatment modalities for medical operable disease regardless of age (8). Lobectomy is widely accepted as the major chance for curing early-stage NSCLC in the elderly (9). However, due to the advantage of increased pulmonary reserve (10), better perioperative outcomes (11), and non-inferior survival outcomes, evidence has been accumulating supporting more limited resection for early-stage NSCLC in elderly patients when compared with lobectomy (12–14). However, almost all the evidence favoring sublobar resection was derived from retrospective studies (12). Moreover, opposing results of inadequate lymph node removal and inferior survival for sublobar resection was reported (15, 16). The only available major trial to address this question was carried out by Lung Cancer Study Group (LCSG) more than two decades ago (17). Although the results were in favor of lobectomy, subsequent studies have questioned their relevance (18, 19). Given the urgency of this health policy question and the fact that the results of two contemporary prospective trials (20, 21) are not currently available, we used the Surveillance, Epidemiology and End Results (SEER)–Medicare cohort to identify patients older than 75 years treated for pathological stage I NSCLC between 2010 and 2015, and investigated the overall survival (OS), lung cancer–specific survival (LCSS), and non-cancer–specific survival (NCSS) of sublobar resection and lobectomy. We sought to determine the comparative effectiveness of lobectomy, segmentectomy, and wedge resection with respect to OS, LCSS, and NCSS, and to explore the factors affecting long-term survival of elderly patients with pathological stage I NSCLC.

## Patients and Methods

### Study Population

This retrospective study was approved by the Ethics Committee of the Peking University First Hospital, and data were retrieved from the latest SEER database using SEER^*^STAT 8.3.5 software. The SEER database contains cases who reside within one of 20 geographic catchment areas that account for approximately 28% of the US population.

We identified patients by using the following inclusion criteria: (1) diagnosed during years 2010 and 2015; (2) pathologically confirmed NSCLC; (3) patients aged ≥75 years at the time of diagnosis; (4) tumor size <4 cm; (5) wedge resection (code 21), segmentectomy (code 22), or lobectomy with or without lymph node removal (codes 30 and 33) were performed; (6) NSCLC as the only primary tumor during the follow-up period. We excluded patients with (1) unknown number of examined lymph nodes, (2) nodal disease or distant metastasis at presentation, (3) follow-up time <3 months, and (4) tumors of main bronchus or overlapping lesions of lung (codes 340, 348, and 349).

### Data Collection

Demographic variables (age, gender, race, and marital status), tumor characteristics (size, histologic type, and site), and treatment information (surgical procedure and number of lymph nodes examined during surgery) were collected. Histologic types of NSCLC were categorized using the second edition of the *International Classification of Disease for Oncology* (ICD-O) into adenocarcinoma (ICD-O codes 8140, 8141, 8250–8323, 8480–8550, and 8572), squamous cell carcinoma (ICD-O codes 8050–8123 and 8562), and other lung cancer, including but not restricted to, spindle cell carcinomas, mucoepidermoid malignancies, neuroendocrine, and mixed malignant tumors (ICD-O codes 8032, 8200, 8230, 8240, 8246, 8430, 8470, 8940, and 8980). Survival time as OS and LCSS were retrieved.

### Survival Outcomes

The primary outcomes were OS and LCSS in months. Patients who were alive at the last available follow-up date were right-censored at this date in survival analysis. The OS rate was calculated from the date of surgery to the date of death due to any cause. The cause-specific survival was defined in the SEER registry as the “cause-specific classification of death” and was classified as “Dead (attributable to this diagnosed cancer),” “Alive,” and “Dead of other cause.” The LCSS rate was calculated from the date of surgery to the date of death due to lung cancer. The NCSS was calculated from the date of surgery to the date of death from causes other than lung cancer.

### Propensity Score Matching and Doubly Robust Estimator

Propensity score matching (PSM) analysis is a method used to minimize the potential bias caused by an existing data set for non-random assignment analysis (22, 23). We used PSM to control the inherent bias intertwined to retrospective cohort study. Propensity scores (PS) were generated by logistic regression on the basis of the patients' potential confounding baseline characteristics, including age, sex, race, marital status, year of procedure, and tumor site and size. Then a 1:1 matched sample was created by matching patients who underwent SLR and lobectomy using caliper width equal to 0.2 of the SD of propensity scores and without replacement. The balance of variables after PSM was tested using Student's *t*-test and χ^2^ test or Fisher's exact test for continuous and categorical data, respectively.

Doubly robust estimation combines a form of outcome regression (i.e., the Cox regression) with a model for the expression (i.e., the propensity score) to estimate the causal effect of an exposure on an outcome (24). In this study, the Cox regression was applied after PSM to ascertain a more reliable causal inference.

### Competing Risk Survival Analysis

A competing risk is an alternative outcome that is of equal or more significant clinical importance than the primary outcome and alters the probability of the outcome of interest (25). Competing risk analyses were carried out to elucidate whether lobectomy can lead to more death unrelated to lung cancer while providing better disease control. The sub-hazards of death caused by lung cancer and death unrelated to lung cancer were calculated using a model developed by Fine and Gray (26), and cumulative incidence functions were plotted.

### Statistical Analysis

The OS and LCSS of patients who underwent lobectomy, segmentectomy, and wedge resection were calculated and compared via Kaplan–Meier method in overall population and in four TNM stage strata in both the full cohorts and the PS-matched cohorts. After univariate Cox proportional-hazards regression analyses, significant variables were entered into a multivariable Cox regression model.

Continuous variables were expressed as mean ± SD and categorical variables were presented as frequencies or percentages. The distribution of continuous variables was analyzed by Student's *t*-test. Categorical variables were compared using χ^2^ test or Fisher's exact test. For all analyses, *p*-values < 0.05 in a two-tailed test were considered to indicate statistical significance. All analyses were performed with STATA/MP 15.1 software (StataCorp LLC, College Station, TX, USA).

## Results

### Patients' Characteristics

A total of 3,345 patients with stage I NSCLC (≤4 cm) were identified. Treatment strategy was as follows: 2,415 (72.2%) lobectomy, 736 (22.0%) wedge resection, and 194 (5.8%) segmentectomy. The median follow-up time for the entire cohort was 28 months (range, 3–71 months). Baseline characteristics of full-scale and two major matched cohorts are summarized in Table 1. Patients who underwent SLR were older, more likely to be of white race, less likely to have extensive lymph node examination, and were more likely to have early-stage tumors (Table 1).

**Table 1:** Characteristics of patients undergoing lobectomy and SLR for lung cancer of pathologic stage I in full cohort and two major propensity matched cohorts.

	**Full cohort (*****N*** **=** **3,345)**			**Matched cohort (924 pairs)**			**Matched cohort lymph node (539 pairs)**		
	**Lobectomy**	**SLR**	***p*-value**	**Lobectomy**	**SLR**	***p*-value**	**Lobectomy**	**SLR**	***p*-value**
	***N* (2,415)**	***N* (930)**		***N* (924)**	***N* (924)**		***N* (539)**	***N* (539)**	
Age (years), mean (SD)	79.0 (3.4)	79.9 (3.8)	<0.001	79.8 (3.7)	79.9 (3.7)	0.469	79.3 (3.5)	79.6 (3.7)	0.172
Year, mean (SD)	2012.4 (1.7)	2012.4 (7.7)	0.873	2012.4 (1.7)	2012.4 (1.7)	0.598	2012.6 (1.7)	2012.6 (1.7)	0.985
Gender, *n* (%)									
Female	1,373 (56.9)	563 (60.54)	0.053	590 (63.85)	559 (60.5)	0.137	353 (65.49)	342 (63.45)	0.484
Male	1,042 (43.1)	367 (39.46)		334 (36.15)	365 (39.5)		186 (34.51)	197 (36.55)	
Race, *n* (%)									
White	2,055 (85.09)	839 (90.22)	<0.001	843 (91.23)	833 (90.15)	0.363	488 (90.54)	483 (89.61)	0.545
Black	144 (5.96)	52 (5.59)		39 (4.22)	52 (5.63)		29 (5.38)	37 (6.86)	
Other	216 (8.94)	39 (4.19)		42 (4.55)	39 (4.22)		22 (4.08)	19 (3.53)	
Marital status									
Married	1,321 (54.7)	478 (51.4)	0.086	453 (49.03)	476 (51.52)	0.306	290 (53.8)	286 (53.06)	0.807
Not Married	1,094 (45.3)	452 (48.6)		471 (50.97)	449 (48.59)		249 (46.2)	253 (46.94)	
Insurance									
Insured	2,253 (93.29)	864 (92.9)	0.612	870 (94.16)	858 (92.86)	0.647	508 (94.25)	500 (92.76)	0.327
Medicaid	161 (6.67)	65 (6.99)		53 (5.74)	65 (7.03)		30 (5.57)	39 (7.24)	
Uninsured	1 (0.04)	1 (0.11)		1 (0.11)	1 (0.11)		1 (0.19)	0 (0)	
Tumor variables									
Location									
Left upper lobe	589 (24.39)	269 (28.92)	<0.001	254 (27.49)	264 (28.57)	0.158	155 (28.76)	163 (30.24)	0.003
Left lower lobe	356 (14.74)	127 (13.66)		129 (13.96)	127 (13.74)		62 (11.5)	61 (11.32)	
Right upper lobe	856 (35.45)	332 (35.7)		349 (37.77)	332 (35.93)		207 (38.4)	197 (36.55)	
Right middle lobe	168 (6.96)	30 (3.23)		46 (4.98)	29 (3.14)		25 (4.64)	5 (0.93)	
Right lower lobe	446 (18.47)	172 (18.49)		146 (15.8)	172 (18.61)		90 (16.7)	113 (20.96)	
Histology									
Squamous	614 (25.42)	275 (29.57)	0.047	239 (25.87)	272 (29.44)	0.160	134 (24.86)	144 (26.72)	0.763
Adenocarcinoma	1,623 (67.2)	586 (63.01)		622 (67.32)	583 (63.1)		369 (68.46)	358 (66.42)	
Other	178 (7.37)	69 (7.42)		63 (6.82)	69 (7.47)		36 (6.68)	37 (6.86)	
Grade									
Grade I	604 (25.01)	220 (23.66)	0.169	255 (27.6)	219 (23.7)	0.068	143 (26.53)	127 (23.56)	0.332
Grade II	1,196 (49.52)	456 (49.03)		431 (46.65)	543 (58.77)		256 (47.5)	266 (49.35)	
Grade III	604 (25.01)	244 (26.24)		235 (25.43)	242 (26.19)		137 (25.42)	138 (25.6)	
Grade IV	11 (0.46)	10 (1.08)		3 (0.32)	10 (1.08)		3 (0.56)	8 (1.48)	
T Stage									
T1	1,610 (66.67)	683 (73.44)	<0.001	695 (75.22)	677 (73.27)	0.338	408 (75.7)	384 (71.24)	0.098
T2	805 (33.33)	247 (26.56)		229 (24.78)	247 (26.73)		131 (24.3)	155 (28.76)	
Number of lymph nodes harvested^†^									
None	82 (3.4)	380 (40.86)	<0.001	27 (2.92)	377 (40.8)	<0.001			
1–3 nodes	362 (14.99)	255 (27.42)		177 (19.16)	254 (27.49)		114 (21.15)	249 (46.2)	<0.001
>4 nodes	1,971 (81.61)	295 (31.72)		720 (77.92)	293 (31.71)		425 (78.85)	290 (53.8)	

*Variables are presented as mean ± SD or n (%). SLR, sublobar resection*.

† *This was not a presurgical variable, therefore not matched*.

### Survival Outcomes

**Figure 1:**
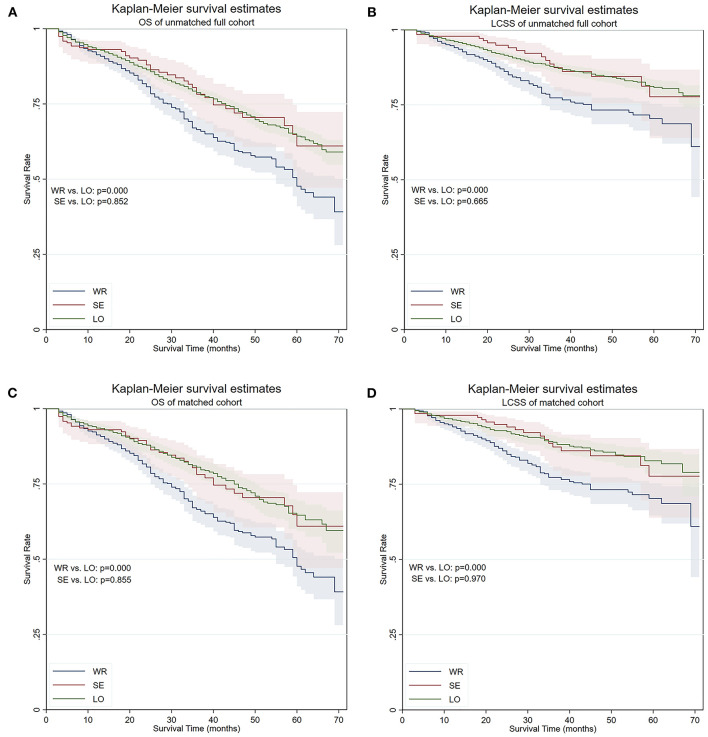
OS and LCSS of the full cohort and PS-matched cohort. **(A)** OS of the full cohort. **(B)** LCSS of the full cohort. **(C)** OS of the PS-matched cohort. **(D)** LCSS of the PS-matched cohort. OS, overall survival; LCSS, lung cancer–specific survival; PS, propensity score; WR, wedge resection; SE, segmentectomy; LO, lobectomy.

The median OS time was 60 months for patients receiving wedge resection, but they were not reached in segmentectomy, lobectomy, and the full cohort. The 5-year OS of patients who underwent wedge resection, segmentectomy, and lobectomy were 47.7, 61.0, and 64.3%, respectively. The 5-year LCSS of patients who underwent wedge resection, segmentectomy, and lobectomy were 70.4, 77.7, and 80.8%, respectively. Although there was no statistical difference in OS (*p* = 0.852) and LCSS (*p* = 0.855) between segmentectomy and lobectomy, significantly worse OS (*p* = 0.000) and LCSS (*p* = 0.000) were noticed in patients who underwent wedge resection when compared with those of lobectomy, and similar results were obtained from the matched cohort (Figure 1). In the matched cohort, advanced age, earlier surgery date, male gender, higher grade, higher T stage, and lesser extensive lymph node dissection were independent risk factors for worse OS, and age, male gender, higher grade, higher T stage, and lesser extensive lymph node dissection were independent risk factors for worse LCSS (Table 2).

**Table 2:** Matched Cox multivariate regression.

	**HR**	**95% CI**	***p*-value**
**Overall survival**			
Surgical procedure (wedge resection, segmentectomy, and lobectomy)	0.913	0.809–1.031	0.143
Age at diagnosis	1.034	1.009–1.059	0.007
Year of surgery	0.916	0.846–0.991	0.029
Gender	1.505	1.245–1.818	<0.001
Tumor grade	1.442	1.268–1.641	<0.0001
T stage	1.300	1.174–1.44	<0.0001
Number of lymph nodes harvested	0.747	0.653–0.855	<0.001
**Lung cancer–specific survival**			
Surgical procedure	0.895	0.758–1.056	0.189
Age at diagnosis	1.033	1.000–1.067	0.047
Year of surgery	0.912	0.823–1.010	0.077
Gender	1.503	1.163–1.943	0.002
Tumor grade	1.759	1.471–2.103	<0.001
T stage	1.334	1.162–1.532	<0.001
Number of lymph nodes harvested	0.672	0.560–0.806	<0.001

**Figure 2:**
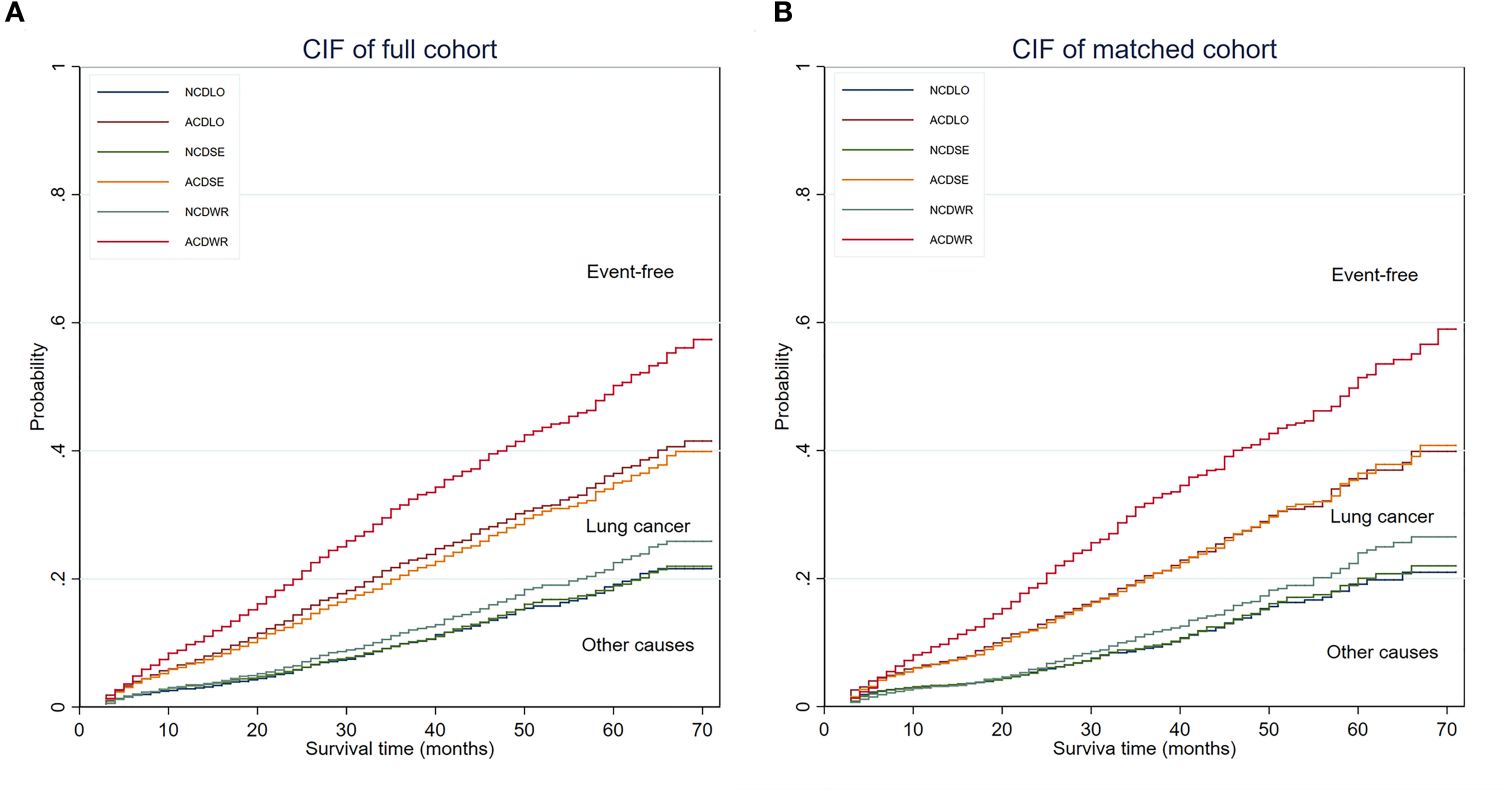
CIF of the full cohort and PS matched cohort. **(A)** CIF of the full cohort. **(B)** CIF of the PS-matched cohort. The area below the non-cancer–related death incidence function was defined as the cumulative incidence of non-cancer–related death, the area between the non-cancer–related death incidence function, and the all-cause death incidence function was defined as the cumulative incidence of lung cancer–related death, and the area above the all-cause death incidence function was defined as the cumulative incidence of being event free. CIF, cumulative incidence function; NCD, non-cancer–related death; ACD, all-cause death; PS, propensity score; WR, wedge resection; SE, segmentectomy; LO, lobectomy.

Competing risks analyses were conducted. In univariate analyses, wedge resection showed worse LCSS than lobectomy, with no significant survival advantage in terms of NCSS, and similar results were obtained from the matched cohort. Segmentectomy seemed to be equivalent to lobectomy in terms of CSS and NCSS in both the unmatched and matched cohorts (Figure 2). In the multivariate competing risks analysis, both wedge resection and segmentectomy showed non-inferiority in LCSS and NCSS to lobectomy in matched cohort (Table 3).

**Table 3:** Multivariate competing risks analyses.

**Variables**	**Sub-hazard ratio**	**95% CI**	***p*-value**
**Lung cancer–specific survival, segmentectomy vs. lobectomy**			
Segmentectomy	1.005	0.620–1.628	0.984
Gender	1.394	0.960–2.025	0.081
Year of surgery	0.859	0.744–0.991	0.037
Tumor grade	2.145	1.684–2.733	<0.001
T stage	1.326	1.053–1.670	0.016
**Non-cancer–specific survival, segmentectomy vs. lobectomy**			
Segmentectomy	1.093	0.667–1.790	0.724
Age	1.052	1.004–1.103	0.034
T stage	1.283	1.048–1.569	0.016
**Lung cancer–specific survival, wedge resection vs. lobectomy**			
Wedge resection	1.226	0.870–1.727	0.245
Age	1.030	0.995–1.066	0.090
Gender	1.396	1.065–1.829	0.016
Year of surgery	0.908	0.820–1.005	0.062
Tumor grade	1.689	1.407–2.028	<0.001
T stage	1.336	1.152–1.551	<0.001
Lymph node harvesting	0.680	0.554–0.834	<0.001
**Non-cancer–specific survival, wedge resection vs. lobectomy**			
Wedge resection	1.249	0.934–1.670	0.134
Gender	1.454	1.084–1.951	0.012
Year of surgery	0.858	0.762–0.967	0.012
T stage	1.170	1.006–1.362	0.042

**Figure 3:**
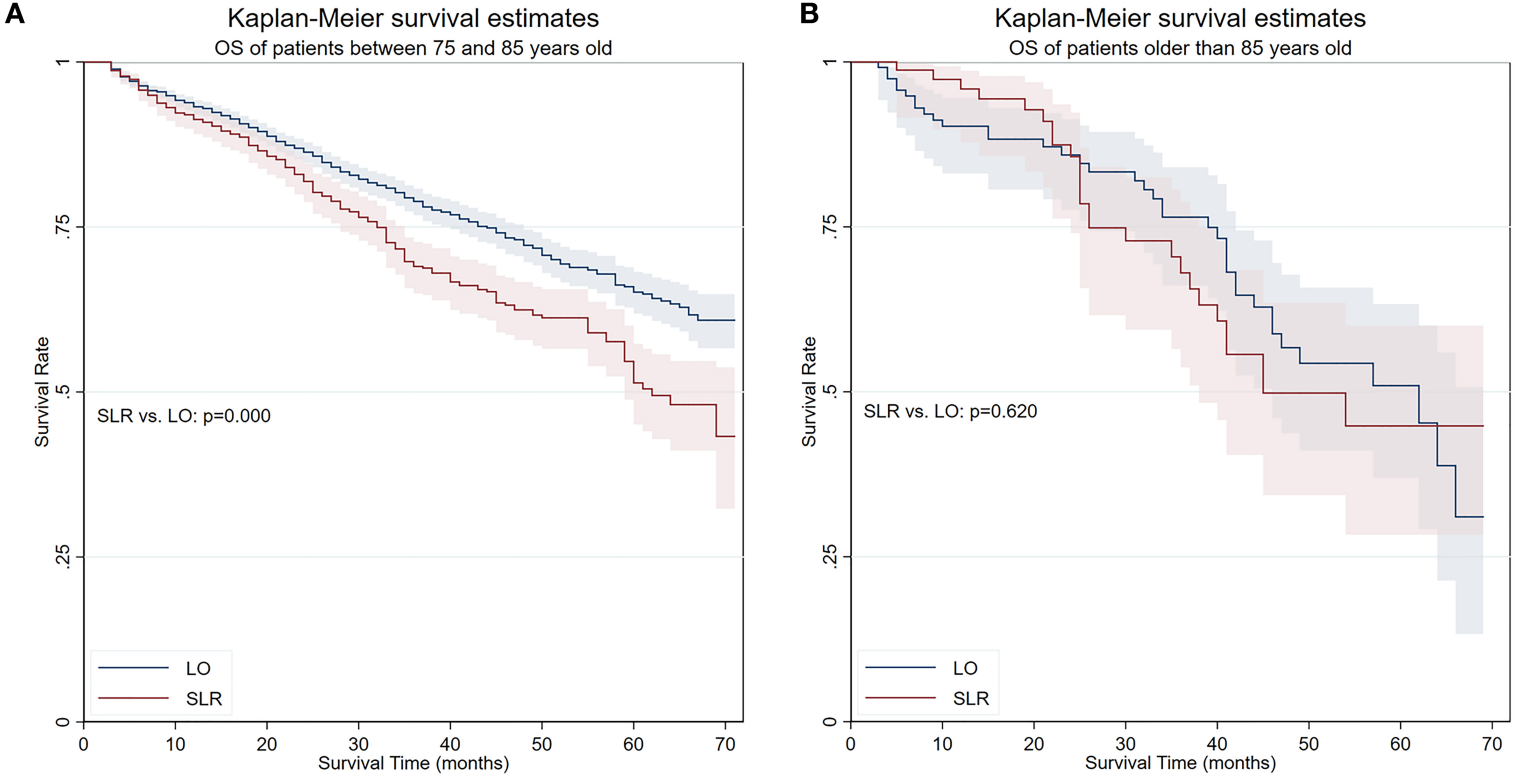
OS of patients in two age groups. **(A)** OS of patients between 75 and 85 years old. **(B)** OS of patients older than 85 years. OS, overall survival; SLR, sublobar resection; LO, lobectomy.

The OS and LCSS were then compared between lobectomy and SLR in different age groups in a “trial and error” fashion. Finally, results showed that the OS and LCSS advantage of lobectomy ceased to exist in patients older than 85 years (Figure 3). A PS matching was performed to longitudinally compare LCSS and NCSS between age groups (patients between 75 and 85 years old and patients older than 85 years). No significant LCSS [sub-hazard ratio (SHR): 1.400, 95% CI 0.797–2.458, *p* = 0.242] and NCSS (SHR: 1.586, 95% CI 0.948–2.654, *p* = 0.079) difference was found by competing risks analyses in this matched cohort.

**Figure 4:**
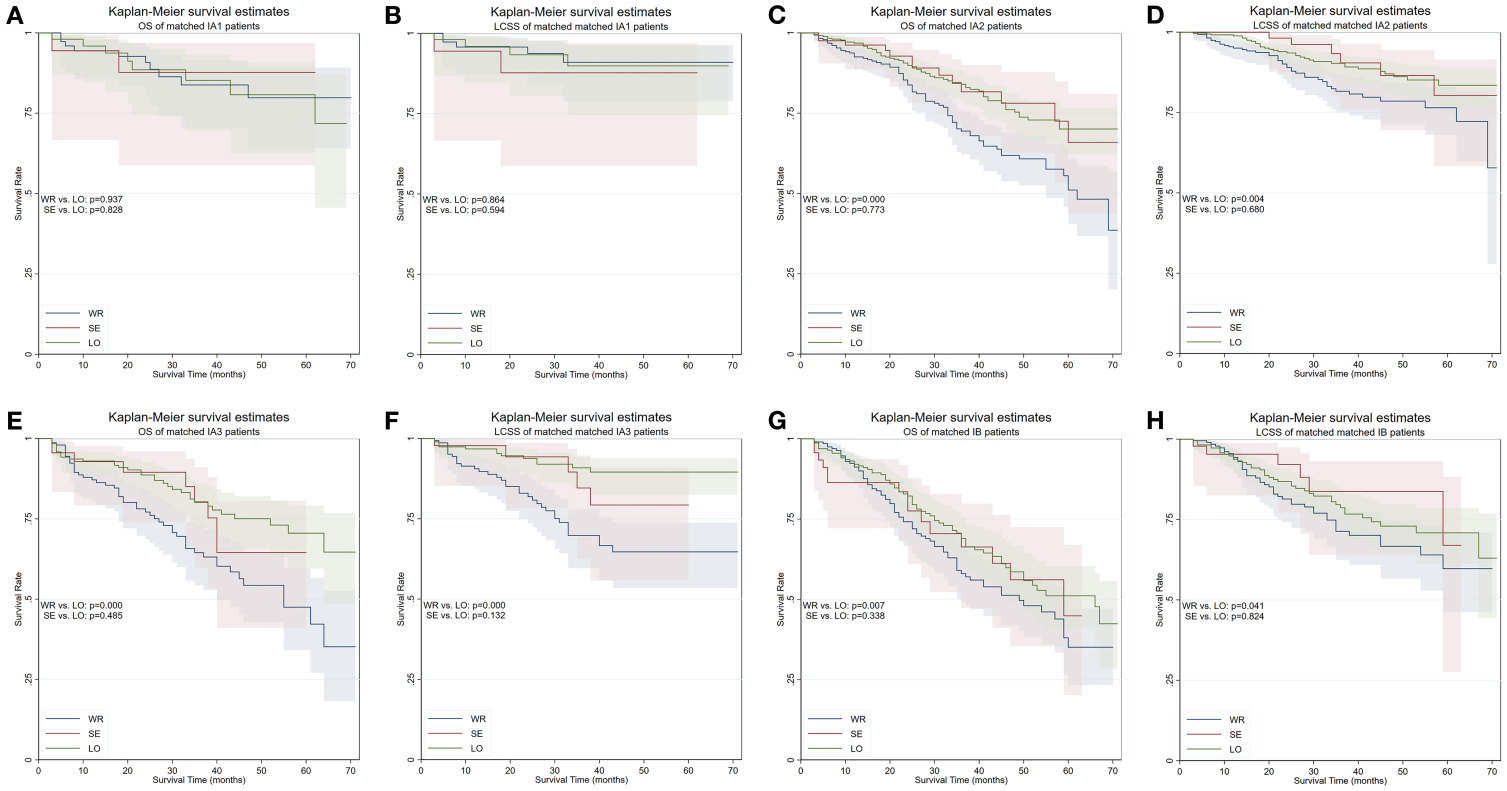
OS and LCSS of PS-matched patients with NSCLC in four TNM stages. **(A)** OS of patients with stage IA1 NSCLC. **(B)** LCSS of patients with stage IA1 NSCLC. **(C)** OS of patients with stage IA2 NSCLC. **(D)** LCSS of patients with stage IA2 NSCLC. **(E)** OS of patients with stage IA3 NSCLC. **(F)** LCSS of patients with stage IA3 NSCLC. **(G)** OS of patients with stage IB NSCLC. **(H)** LCSS of patients with stage IB NSCLC. OS, overall survival; LCSS, lung cancer–specific survival; PS, propensity score; WR, wedge resection; SE, segmentectomy; LO, lobectomy.

The patients were stratified by pathological stage and four matched strata were generated. No statistical survival difference was observed between segmentectomy and lobectomy in both matched and unmatched cohorts at four strata (Table 4, Figure 4). Wedge resection showed significant worse OS and NCSS in IA2, IA3, and IB patients when compared with lobectomy (Table 4, Figure 4).

**Table 4:** Stratified univariate Cox regression analyses in matched cohort.

**T stage**	**Wedge resection vs. lobectomy**			**Segmentectomy vs. lobectomy**		
	**HR**	**95% CI**	***p*-value**	**HR**	**95% CI**	***p*-value**
**Overall survival**						
IA1	1.094	0.431–2.777	0.851	1.346	0.294–6.166	0.702
IA2	1.836	1.317–2.560	<0.001	0.956	0.526–1.737	0.883
IA3	2.042	1.325–3.149	0.001	1.192	0.572–2.485	0.640
IB	1.587	1.132–2.225	0.007	1.316	0.747–2.316	0.342
**Lung cancer–specific survival**						
IA1	1.298	0.324–5.195	0.713	3.172	0.577–17.44	0.184
IA2	1.804	1.147–2.838	0.011	0.818	0.343–1.948	0.650
IA3	3.433	1.847–6.383	<0.001	1.598	0.575–4.437	0.368
IB	1.571	1.014–2.435	0.043	0.907	0.382–2.153	0.824

**Figure 5:**
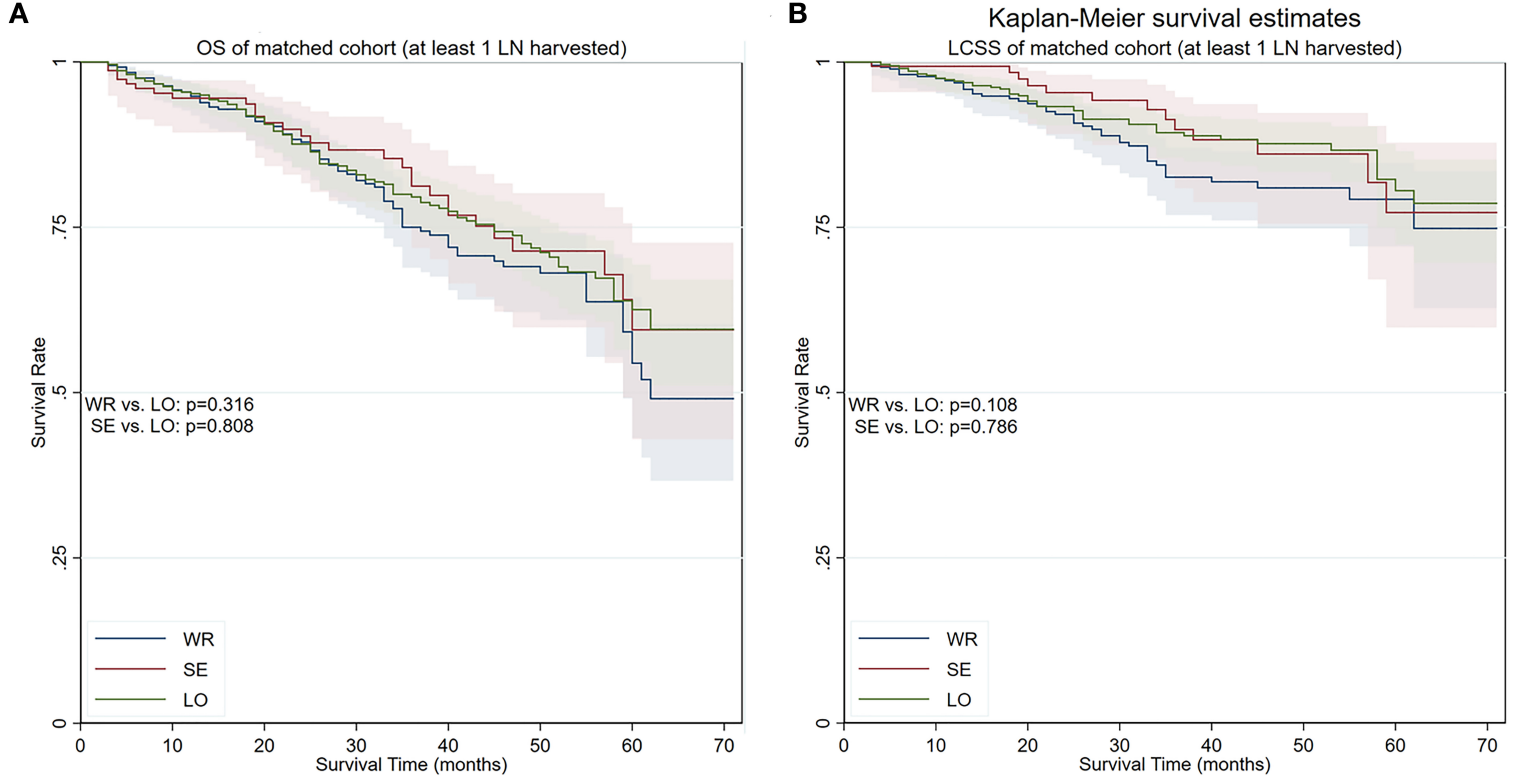
OS and LCSS of PS-matched patients with at least one lymph node detected during the procedure. **(A)** OS of PS-matched patients with at least one lymph node detected during the procedure. **(B)** LCSS of PS-matched patients with at least one lymph node detected during the procedure. OS, overall survival; LCSS, lung cancer–specific survival; PS, propensity score; WR, wedge resection; SE, segmentectomy; LO, lobectomy.

We conducted yet another PS matching and yielded a cohort containing 539 matched pairs of patients who had one or more lymph nodes examined during surgery (Table 1). The cohort contained 539 (50.0%) lobectomy (5-year OS 66.6%, 5-year LCSS 80.5%), 153 (14.2%) segmentectomy (5-year OS 60.8%, 5-year LCSS 77.2%), and 386 (35.8%) wedge resections (5-year OS 54.5%, 5-year LCSS 79.2%). While segmentectomy remained similar to lobectomy in terms of both OS (*p* = 0.804) and LCSS (*p* = 0.786) in this cohort, the differences in terms of OS (*p* = 0.316) and LCSS (*p* = 0.108) ceased to exist between wedge resection and lobectomy (Figure 5).

**Figure 6:**
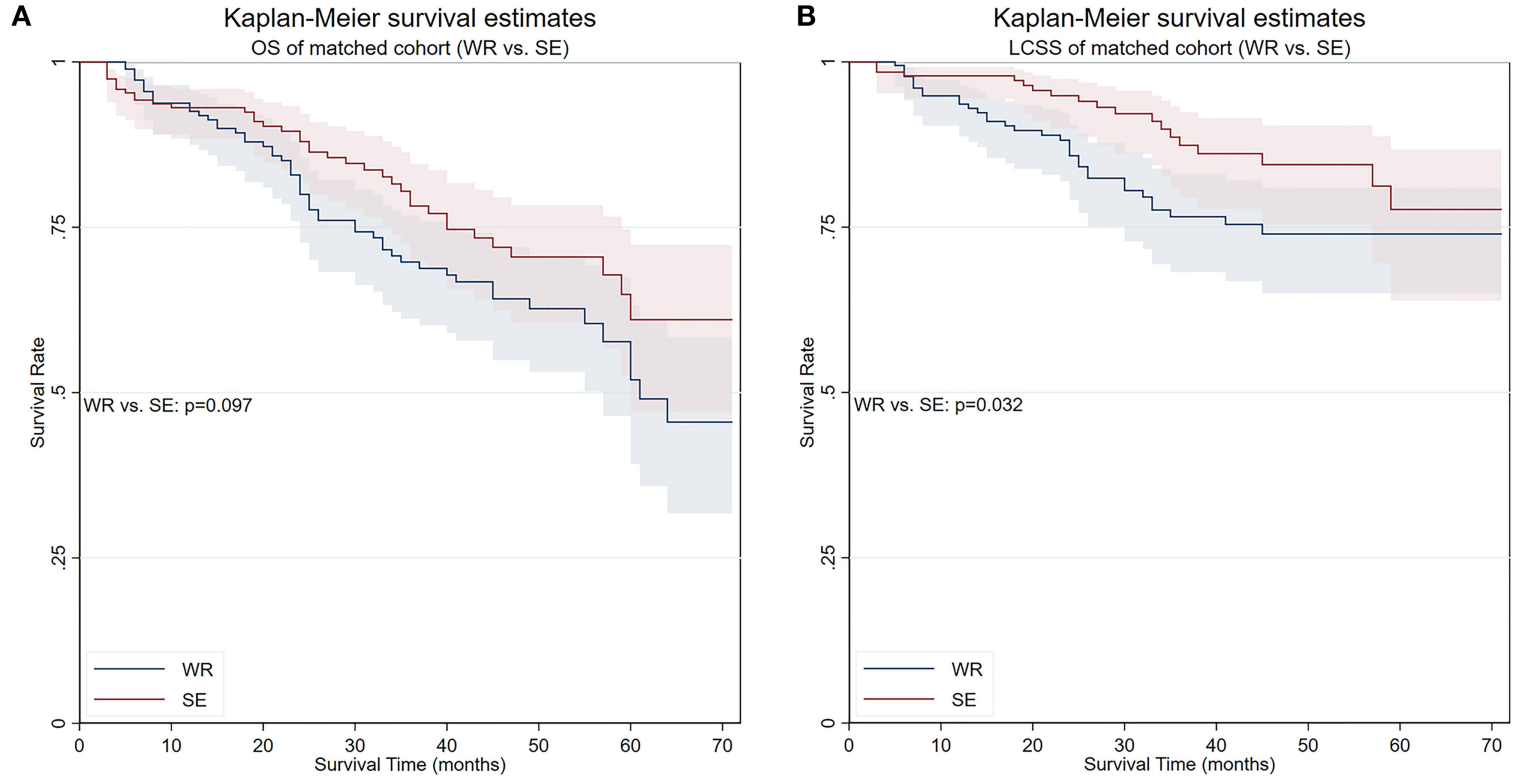
OS and LCSS of PS-matched patients who underwent either wedge resection or segmentectomy. **(A)** OS of PS-matched patients who underwent either wedge resection or segmentectomy. **(B)** LCSS of PS-matched patients who underwent either wedge resection or segmentectomy. PS, propensity score; WR, wedge resection; SE, segmentectomy; LO, lobectomy.

To elucidate whether there is a survival difference between wedge resection and segmentectomy, another PS matching was performed. The balances were achieved except for scale of lymph nodes resection. Survival analyses conducted in the matched cohort showed that the OS of patients who underwent segmentectomy was marginally superior to wedge resection (5-year survival 61.0 vs. 47.7%, respectively, *p* = 0.097), while the LCSS was significantly better (5-year survival 77.7 vs. 74.0%, respectively, *p* = 0.032) (Figure 6). Competing risks analysis confirmed the LCSS superiority of segmentectomy (SHR: 0.537, 95% CI 0.307–0.938, *p* = 0.029).

## Discussion

In the current study, we found out that in patients ≥75 years old who underwent SLR or lobectomy for stage I NSCLC: (1) comparing to wedge resection, lobectomy offered superior OS and LCSS without affecting long-term NCSS, although its OS advantage disappeared when patients were over 85 years old; (2) lobectomy held both the OS and LCSS superiority to wedge resection, not to segmentectomy, in the full cohort and most T-stage stratified cohorts, however, the survival difference ceased to exist when at least one lymph node was detected during surgery; (3) segmentectomy provided marginally better OS and significant better LCSS when compared with wedge resection.

Lobectomy was deemed the standard curative procedure for early-stage NSCLC since the trial held by LCSG (17). However, at least partially due to the marginal survival advantage (*p* = 0.08) reported by that study, surgeons frequently hesitated to perform lobectomy in patients with advanced age, comorbidities, or limited pulmonary function reserve. Instead, SLR is often offered as an alternative, with the belief that a more abundant postoperative capacity would benefit the overall long-term survival. This trend is more striking in elderly patients (13, 27). Nonetheless, the functional superiority of SLR has yet to be established (28). In addition, our study showed that the NCSS of patients who underwent lobectomy was non-inferior to segmentectomy, and superior to wedge resection, suggesting that lobectomy did not increase the burden of postoperative long-term NCSS in elderly patients. Still, because some of the advantages of SLR in short term were advocated by several studies (11, 14, 29), as well as the non-inferiority of SLR in comparison with lobectomy was suggested (13, 27, 30–32), SLR should be considered in carefully selected elderly patients.

In the perspective that life expectancy shortens during the progress of the aging process, it is assumed that the survival benefit of lobectomy over SLR will be diminished by the increasing risk of mortality from causes other than lung cancer. Similar to Mery et al. (27), our results suggested that the OS difference between patients receiving SLR and lobectomy receded in older patients. Nonetheless, unlike the cut-point found by the aforementioned study, which is 75 years old, the said cut-point was 85 years old in our study. This shifting could be stemmed from the technological advancement in the field of surgical oncology, as well as a sign of development in general health care services.

Regarding stratified survival outcomes, out results agreed with the majority of former studies' conclusion that sublobar resection was equivalent to lobectomy in terms of survival in patients with smaller sized tumor (13, 33). A detailed stratified comparison in elderly patients is, unfortunately, absent. As IASLC (International Association for the Study of Lung Cancer) asserted that from 1 to 5 cm every centimeter count (34), we scrutinized the survival outcomes in such style. Matched survival analyses showed that, despite the equivalent survival of lobectomy and segmentectomy throughout the whole four strata, wedge resection shared the said similarity only in pathologic IA1 stage. This result agreed with previous studies that focused on the effect of different surgical procedures in younger NSCLC patients, indicating that wedge resection may not be an appropriate procedure for patients with NSCLC other than IA1 stage even at advanced age.

Another significant result observed in this study is the confirmation of the role of lymphadenectomy. It is well established that the extent of lymph node dissection was correlated, if not with both better staging and survival according to Halsted view, with a more accurate staging at least, by the Cady–Fisher view (35). Stiles et al. demonstrated that SLR resulted in fewer lymph node resections and was associated with inferior survival when compared with lobectomy in patients with tumor size ≤5 cm (15, 16). In our study, the number of lymph nodes detected during surgery was correlated with survival, as the OS and LCSS differences diminished in the propensity score–matched group when at least one lymph node was examined, and the said correlation did not cease to exist until the age exceeded 85 years. Considering the non-negligible reported occult nodal metastases rate in clinical stage IA NSCLC, which ranges from 4 to 12% (36, 37), the correlation between the number of lymph nodes detected during surgery and survival in our study was possibly the effect of the “Will Rogers” phenomenon brought by the undetected stage shafting effect resulting from the inadequate intraoperative lymph node evaluation. Thus, it is appropriate to perform lymph node evaluation provided that there is no evidence for non-invasiveness even in the resection of stage IA1 NSCLC. The underlying mechanism that the effect of number of lymph nodes on long-term survival diminishes with age is currently unclear. Considering the equivalent NCSS between the two age groups, our study resonated with the results obtained by Han-Yu Deng et al. (38) that age could be an independent predictor of lymph node metastasis, which indirectly affected the oncological effects of lymph node harvesting in elderly patients. This question is expected to be solved in more extensive and specific research in the future.

Taking a closer scrutiny to the SLR, it is not hard to assume that a survival difference should lay between wedge resection and segmentectomy, due to the distinct surgical approach to hilar structures and, possibly more important, lymph node removal. Unfortunately, current evidence is inadequate to determine the said hypothesized survival difference due to the absence of relevant randomized controlled trials (RCTs). Although a considerable amount of retrospective studies have suggested that segmentectomy is superior to wedge resection regarding long-term survival (39–41), there is evidence to the contrary that wedge resection is similar to segmentectomy (42, 43). Our results suggested that segmentectomy is superior to wedge resection in terms of LCSS in patients over 75 years old, although it may not have an OS advantage over wedge resection. The origin of the said superiority could be derived from the different extent of lymphadenectomy, as well as the intrinsic difference between the surgical procedures.

The study has several limitations. First, it is more ideal that the clinically staged patients be analyzed in addition to pathologically staged patients. Unfortunately, clinical staging information was not available in the SEER database. Second, although the PS matching could partially offset the potential confounding factors, it is at the expense of the size of the targeted cohort. Our statistical power could hence be inadequate to prove non-inferiority. Furthermore, it is the nature of PS matching that only the recorded factors could be used to fashion a simulated randomization. Taking into account that the data of smoking history, performance status, and pulmonary function were not available, we cannot stress enough that our results may be affected by uncontrolled bias, which made our conclusions less convincing. For example, lobectomy would theoretically positively affect LCSS while negatively affecting NCSS due to a more radical resection in comparison with SLR; however, elucidation of the said effects requires adequate control of patient selection bias that derives from the different preoperative functional states of patients, which is unavailable in our study. Finally, it is well-established that retrospective evaluation of data from administrative database is not a substitute for randomized trials. However, it is unlikely that a RCT could be conducted to answer the specific clinical question. Despite the inherent biases that are intertwined with the study's retrospective nature, we do believe that, by controlling available demographic and tumor-related factors with PS matching, the biases can be minimized to the best extent possible.

## Conclusions

Our study demonstrates that in the treatment of stage I NSCLC, lobectomy does not negatively affect NCSS in comparison with SLR in patients >75 years old and should be considered the “gold standard” for the treatment of NSCLC; nonetheless, wedge resection with lymphadenectomy may be a non-inferior alternative in elderly patients who are >85 years old and in elderly patients with pathologic stage IA1 NSCLC. In addition, segmentectomy and lobectomy seem to be comparable procedures for elderly patients with pathological stage I NSCLC. Lymphadenectomy should be conducted as part of SLR in elderly patients younger than 85 years unless there is adequate evidence that it is a minimally invasive tumor. Further studies should be performed to elucidate the effectiveness of SLR in elderly patients with stage I NSCLC.

## Data Availability Statement

The raw data supporting the conclusions of this article will be made available by the authors, without undue reservation.

## Ethics Statement

The studies involving human participants were reviewed and approved by the Ethics Committee of the Peking University First Hospital. The patients/participants provided their written informed consent to participate in this study.

## Author Contributions

XZ and GL participated in research design, data analysis, and wrote the paper. XZ and JL participated in research design and data analysis. All authors contributed to the article and approved the submitted version.

## Conflict of Interest

The authors declare that the research was conducted in the absence of any commercial or financial relationships that could be construed as a potential conflict of interest.
